# The Evolution of BRAF Activation in Non-Small-Cell Lung Cancer

**DOI:** 10.3389/fonc.2022.882940

**Published:** 2022-07-13

**Authors:** Longyao Zhang, Linpeng Zheng, Qiao Yang, Jianguo Sun

**Affiliations:** ^1^ Cancer Institute, Xinqiao Hospital, Army Medical University, Chongqing, China; ^2^ Department of Ultrasound, The 941Hospital of the Chinese People's Liberation Army (PLA) Joint Logistic Support Force, Xining, China

**Keywords:** BRAF activation, EGFR mutation, non-small cell lung cancer, targeted therapy, acquired resistance, immune checkpoint inhibitors

## Abstract

Non-small-cell lung cancer (NSCLC) is the most common subtype of lung cancer, of which approximate 4% had BRAF activation, with an option for targeted therapy. BRAF activation comprises of V600 and non-V600 mutations, fusion, rearrangement, in-frame deletions, insertions, and co-mutations. In addition, BRAF primary activation and secondary activation presents with different biological phenotypes, medical senses and subsequent treatments. BRAF primary activation plays a critical role in proliferation and metastasis as a driver gene of NSCLC, while secondary activation mediates acquired resistance to other targeted therapy, especially for epidermal growth factor tyrosine kinase inhibitor (EGFR-TKI). Treatment options for different activation of BRAF are diverse. Targeted therapy, especially two-drug combination therapy, is an important option. Besides, immune checkpoint inhibitors (ICIs) would be another option since BRAF activation would be a positive biomarker of tumor response of ICIs therapy. To date, no high level evidences support targeted therapy or immunotherapy as prioritized recommendation. After targeted therapy, the evolution of BRAF includes the activation of the upstream, downstream and bypass pathways of BRAF. In this review, therapeutic modalities and post-therapeutic evolutionary pathways of BRAF are discussed, and future research directions are also provided.

## Introduction

Lung cancer is the leading reason of cancer death worldwide, accounting for 18% of all and non-small-cell lung cancer (NSCLC) is the most common subtype of lung cancer (1). With the development of precision medicine, especially next-generation sequencing (NGS) technology and circulating tumor DNA (ctDNA) technology, targeted therapy has replaced platinum-based chemotherapy as the first-line treatment for NSCLC patients with driver gene mutations (2, 3). More and more driver genes have been found in NSCLC, among which activated BRAF proto-oncogene accounts for approximate 4% (4, 5). BRAF mutant tumors are characterized as an aggressive histologic pattern with micropapillary features, and indicates a poor prognosis.

This review provides a comprehensive overview of characteristics, treatment modalities, and outcomes for NSCLC patients with different BRAF mutations. The pathways of activation and evolution of BRAF are divided into primary and secondary mutations. And different BRAF types have different clinical, biological and pathological features. The mechanism of acquired resistance and subsequent evolution of BRAF activation and the strategies after resistance are also discussed.

## A Brief History of Braf Signaling

**Figure 1:**
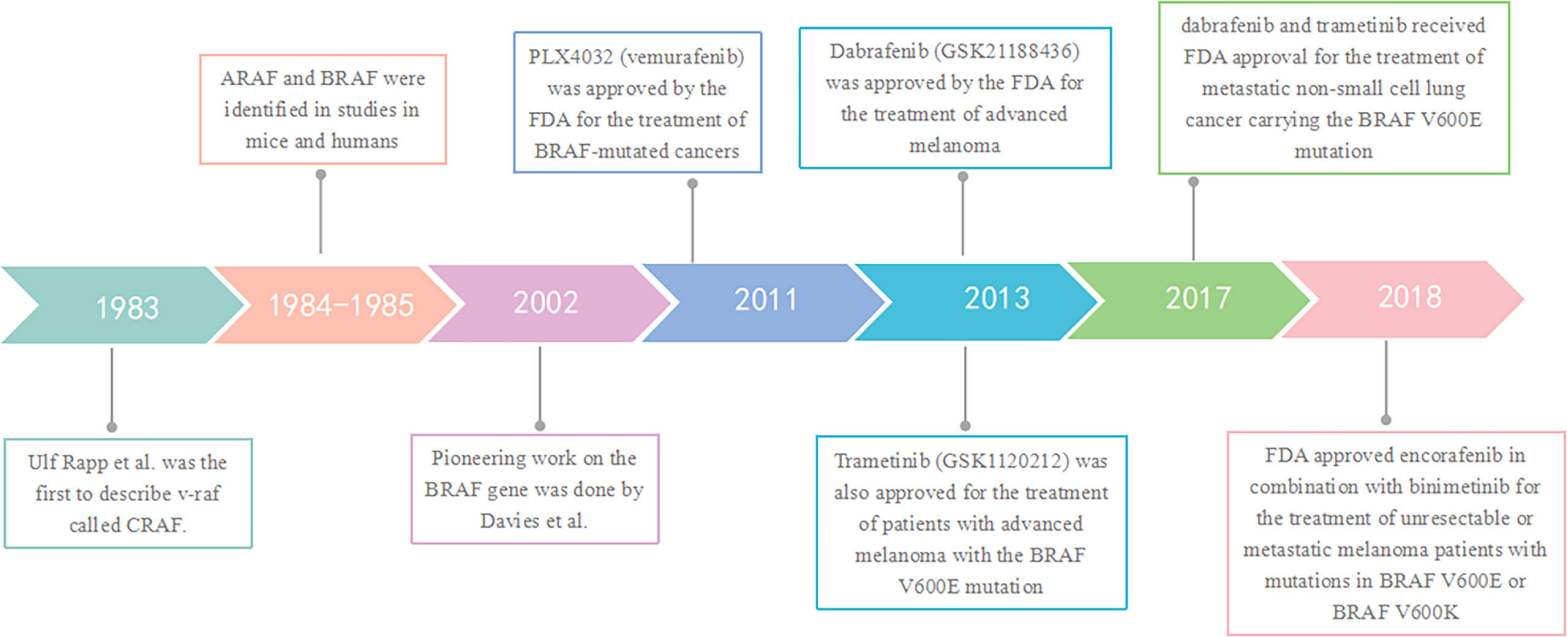
Timeline of key events in BRAF signaling research.

The RAF kinase has been closely and inextricably linked to cancer since 1983, when v-raf was first described by Ulf Rapp et al. (6) This is a murine retroviral oncogene with a mammalian cell homolog, called CRAF. And in 1984-1985, two CRAF-related genes were identified in studies in mice and humans: ARAF and BRAF (7, 8). In 2002, following the pioneering work of Davies et al. (9) on the BRAF gene, a number of studies have clarified the specific implications of BRAF mutations in lung cancer (10, 11). In 2011, following the results of a phase III trial (BRIM-3), the FDA approved the first drug targeting BRAF-mutated cancers, PLX4032 (vemurafenib) (12). Two years later, based on the results of the Phase III trial (NCT01227889), another targeted agent against BRAF mutations, Dabrafenib (GSK21188436), was also approved by the FDA for the treatment of advanced melanoma (13). In the same year, Trametinib (GSK1120212) was also approved for the treatment of patients with advanced melanoma with the BRAF V600E mutation (14). In 2017, dabrafenib and trametinib received FDA approval for the treatment of metastatic non-small cell lung cancer carrying the BRAF V600E mutation (15). The next year, the FDA approved encorafenib in combination with binimetinib which is an anti-MEK1/2 protein kinase inhibitor for the treatment of unresectable or metastatic melanoma patients with mutations in BRAF V600E or BRAF V600K based on a Phase III randomized, active-controlled, open-label, multicenter trial (COLUMBUS) (16). (**Figure 1**)

## The Activation of Braf

### The Primary Activation of BRAF

BRAF is a mammalian cytosolic serine/threonine kinase that belongs to the rapidly accelerated fibrosarcoma (RAF) kinase family (ARAF, BRAF, CRAF), which uses the mitogen-activated protein kinase (MAPK) pathway to transmit signals downstream of RAS (17). The primary activation of BRAF comprises of BRAF classic mutations, BRAF rare mutations, BRAF fusion and amino acid insertion, etc. (17, 18). Different types of BRAF classical mutations have different clinical, pathological and biological characteristics. Studies have found that the occurrences of BRAF V600E mutations were not associated with age, tumor size, lymph node status, tumor stage and BRAF V600E mutation is more common in female lung adenocarcinoma, but very rare in male or squamous cell carcinoma (19). Besides, BRAF non^-^V600E mutations were prone to be found in smokers. Their occurrences were not associated with clinicopathological parameters or had no impact on prognosis. When treated with platinum-based chemotherapy, NSCLC patients with BRAF V600E mutation had a tendency of shorter progression-free survival (PFS) than those with BRAF non-V600E mutations, and the clinical outcomes between patients with BRAF mutation-positive and wild-types were similar, suggesting that BRAF mutations were not sensitive to chemotherapy.

The BRAF primary classic mutations process that leads to tumors can be broadly divided into three categories. Compared to wild-type, Class I (BRAF V600 mutation) increases 500-700 times kinase activity and activates downstream MAPK cascade pathways through activating monomers in a non-RAS dependent form and transcription factors. Class II and class III are mainly BRAF non-V600 mutations. Class II mutants have moderate kinase activity and can transmit signals through RAS independent constituent dimer to activate MEK1, which in turn activate ERK1/2, ultimately promote cell growth and infinite reproduction. Unlike class II mutants, class III mutants have no or little kinase activity, relying on RAS generates upstream signals that induce class III mutants to signal in the form of dimers (17, 20, 21). In general, class I and class II BRAF mutants can be independent of RAS signals and inhibit the negative feedback of ERK signals. In addition, class I BRAF mutants transmit signals in the form of active monomer, while class II and III BRAF mutants in the form of dimer, and the final signal transduction leads to the continuous activation of MAPK.

In addition to the three types of BRAF mutations, there are other forms of mutations that lead to over-activation of the pathway and ultimately the development of tumors. For example, BRAF in-frame deletions are mutually exclusive with RAS mutations, and these mutations can continuously transmit signals to activate the MAPK pathway by forming BRAF homologous dimers (22). Another study has also demonstrated that BRAF internal deletion is a mechanism of acquired drug resistance to BRAF/MEK inhibitors (23).

BRAF fusion has also been reported to be associated with tumorigenesis and progress. It was reported that BRAF fusion could cause the deletion of n-terminal inhibitory domain and activate downstream MAP kinase signal through recruiting CRAF protein to form a dimer (24, 25). In addition, a study reported that two melanoma cases whose pathogenesis was similar to BRAF fusion leading to tumorigenesis, but different from BRAF fusion, these two cases leaded to the over-activation of the pathway through the loss of BRAF inhibitory domain caused by chromosomal translocations of BRAF oncogene (26).

Amino acid insertions were found at position 599 of the BRAF codon, which is rare in the BRAF primary gene alteration. It is speculated that this may be related to the increase of kinase activity caused by changes in the spatial structure of the P ring (18).

NGS technology has revealed better comprehensive understanding of the gene mutations in various tumors. For BRAF, more and more co-mutations have been found between BRAF and other genes, which also indicates that the branching cloning process occurs at the early stage of tumor evolution, which leads to the generation of BRAF co-mutations. According to literature reports, BRAF can co-occur with KRAS mutation (27, 28), NRAS mutation (29), PTEN mutation (30, 31), MEK2 mutation (32), PIK3CA mutation (33) and other gene mutations. And most of these BRAF co-mutations occur in melanoma and lung cancer, but also found in other tumors.

### The Secondary Activation of BRAF

The secondary activation of BRAF includes BRAF classic mutations, BRAF fusion and rearrangement, which are mainly acquired resistance to EGFR-TKI (25). Osimertinib has been prior recommended to the resistance caused by first- and second-generation EGFR-TKIs (34). But it will inevitably cause acquired resistance. It has been reported that the underlying mechanisms of acquired resistance to third-generation EGFR-TKIs include activation of parallel pathways, such as mutations of BRAF or other genes, and rearrangement of resistant genes, such as fusions of BRAF or other genes (25). The mutation and fusion mechanism of BRAF induced by EGFR-TKI resistance constitute an important part of BRAF gene evolution, and different treatment schemes have been explored for different types of BRAF evolution. BRAF rearrangement accounts for 4.4% of BRAF changes in NSCLC, and BRAF fusion is a form of BRAF rearrangement. Four cases were reported that BRAF fusion was a mechanism of EGFR-TKI acquired resistance in EGFR mutant lung adenocarcinoma (25).

Three cases revealed BRAF V600E mutation may be the mechanism for acquired crizotinib resistance after ROS1 rearrangement in NSCLC, two of them had acquired ROS1 rearrangement co-existing with BRAF V600E (35, 36). In another patient, ROS1 rearrangement was lost during treatment, leaving only the BRAF V600E mutation (37). Through single circulating tumor cell (CTC) sequencing, researchers found that patients with ALK mutation developed acquired drug resistance after ALK-TKIs therapy (27). And the mechanism of ALK-TKIs resistance mainly included mutations of RTK-KRAS pathway and TP53 pathway independent of ALK pathway. In the RTK-KRAS pathway, BRAF mutations accounted for 6.2% of the RTK-KRAS pathway (38). In addition, studies showed that BRAF mutation and BRAF fusion were secondary to adagrasib therapy in patients with KRAS G12C mutation (39).

## The Therapy of Braf Activation

The treatment of BRAF mutation is mainly divided into two types, one is BRAF V600 mutation and the other is BRAF non-V600 mutation. BRAF V600 accounts for approximately 50% of BRAF mutation, and is more aggressive, and it occurs by mutation of glutamate into valine at position 600 of exon 15 (40). BRAF V600 develops by the previously described Class I mutation that activates the pathway in RAS independent monomer form. The other type of BRAF non-V600 mutation is mainly the previously described Class II and III BRAF mutations, which develop from signaling to downstream molecules in the form of dimers (17). The class II BRAF mutant is divided into class IIa within the activation segment and class IIb within the glycine-rich p-loop (20). The different structure, mechanism of occurrence and development leads to different treatment modalities for BRAF V600 and BRAF non-V600 mutations. On the other hand, current targeted drugs are mainly targeted at BRAF V600E, while there is no specific treatment modality for BRAF non-V600E.

### Targeting BRAF V600 Mutation

BRAF V600 mutations including V600E, V600K, V600D and other subtypes, among which V600E is the most common subtype. The initial treatment for BRAF V600 mutation was monotherapy and FDA approved the first successful therapy targeting BRAF mutant melanoma called vemurafenib, an oral small molecule inhibitor of BRAF V600 mutations in 2011 (41). The evidence came from a histology-independent, flexible, early phase II “basket” study of vemurafenib in patients with non-melanoma cancers harboring BRAF (42). In this study, the objective response rate (ORR) was 42% (95% confidence interval [CI], 20 to 67%) and the median PFS was 7.3 months (95% CI, 3.5 to 10.8 months). The 12-month rate of PFS was 23% (95% CI, 6 to 46%) and the preliminary 12-month overall survival (OS) rate was 66% (95% CI, 36 to 85%). The most common adverse event was nausea. Vivek Subbiah et al. (43) explored whether BRAF V600E mutations in NSCLC were sensitive to vemurafenib or not. The results turned out that among sixty-two NSCLC patients with BRAF V600 mutation, the overall ORR was 37.1% (95% CI, 25.2 to 50.3%), and 37.5% (95% CI, 8.5 to 75.5%) in previously untreated patients, and 37.0% (95% CI, 24.3 to 51.3%) in previously treated patients. The median PFS was 6.5 months (95% CI, 5.2 to 9.0 months), and the median OS was 15.4 months (95% CI, 9.6 to 22.8 months). Vemurafenib had a similar safety profile in studies focused on melanoma patients. Furthermore, the French National Cancer Institute (INCA) conducted a trial to assess the efficacy and safety of vemurafenib in cancers with various BRAF mutations (44). Among 118 NSCLC patients, 101 of them presented with a BRAF V600E mutation and 17 with BRAF non-V600 mutations. In the BRAF V600 cohort, the ORR was 44.9%, the median PFS was 5.2monthes (95% CI: 3.8 to 6.8%), and the OS was 10 months (95% CI, 6.8 to 15.7 months). The results indicated that vemurafenib is beneficial to NSCLC patients with BRAF V600E mutation.

By inhibiting BRAF V600E kinase activity, dabrafenib resulted in decreased phosphorylation of MEK and ERK, inhibition of cell proliferation, and ultimately G1 cell cycle arrest and cell death (45). In a phase II, multicenter, nonrandomized, open-label study, 84 advanced NSCLC patients with BRAF V600E mutation showed dabrafenib had some active killing effect, though the effect was limited. The adverse events were mainly skin-related, but these adverse events were tolerable (46).

A study had demonstrated that the acquire resistance to BRAF inhibitors was largely caused by reactivating the MAPK signaling pathway (47). Trametinib is a MEK1/2 inhibitor which blocks MEK1/2 kinase activity and prevents RAF-dependent MEK phosphorylation (48). A phase II, multicenter, non-randomized, open-label study assessed the efficacy of the combination of trametinib and dabrafenib, among previously treated or untreated metastatic NSCLC patients with BRAF V600E mutation. All patients were divided into three cohorts. In cohort B, 57 patients were enrolled and resulted in an ORR of 63.2%, disease control rate (DCR) of 79%, median PFS of 9.7 months (95%CI: 6.9-19.6) and 37 patients (65% [95% CI 51–76]) achieved 6-month PFS and median duration of response was 9·0 months ([95% CI 6·9–18·3]. The median OS data are immature, but 47 (82%) of 57 patients were alive at 6 months. The most common adverse event is pyrexia in 26 patients (46%) (49). The results of cohort C of this phase II study demonstrated promising results with ORR of 64% and DCR of 75%,the median PFS of OS 10.9 months and OS of 24.6 months, which was slightly better than in the previously treated cohort (cohort B) of this trial (50). In addition, the side effect profile was mostly similar to that of cohort B, BRAF-MEK combination therapy (dabrafenib plus trametinib) demonstrated tolerability and efficacy in a recent phase II clinical trial and in light of these promising results, combination dabrafenib and trametinib was approved by the US FDA for patients with metastatic melanoma and BRAF V600E mutation. Moreover, a real-life cohort of patients with BRAF mutant advanced NSCLC shows that treatment with BRAF inhibitors and MEK inhibitors in BRAF V600E tumors is associated with ORR of 67%, median PFS of 5.5 months, and median OS since treatment initiation of 9.5 months, which indicate the combination of BRAF inhibitors and MEK inhibitors is clearly superior to monotherapy with a BRAF inhibitors (51). In addition, the incidences of pyrexia and myelosuppression are higher with combination therapy than with monotherapy.

NSCLC patients with EGFR mutation could develop BRAF V600E mutation after acquiring resistance to targeted therapy. Given the secondary activation of BRAF, Huang et al. (52) proposed a strategy of combination of dabrafenib, trametinib and osimertinib, and the patient achieved long-term control of the disease. Another study also demonstrated that the combination of dabrafenib, trametinib and osimertinib was effective to NSCLC patient who developed a BRAF V600 mutation after EGFR-TKI resistance. In addition, the adverse events could be controlled by reducing the dose (53). In another experiment, the treatment of patient also demonstrated that the BRAF inhibitor encorafenib inhibited MEK signaling but had no significant effect on ERK phosphorylation, while the combination of encorafenib and osimertinib significantly reduced MEK and ERK phosphorylation and cell growth (54). In addition, in the review of 7 additional patients who were also reported to be treated with combined therapy of dabrafenib, trametinib and osimertinib, all patients obtained extended PFS and clinical benefit (54–57). In summary, NSCLC patients harbored secondary BRAF V600E mutations because of acquired resistance to EGFR-TKI could benefit from the combination with EGFR-TKI (e.g., osimertinib) and FDA-approved two-drug therapy (e.g., dabrafenib, trametinib).

In 3 patients with secondary activation of BRAF V600E, two patients had both ROS1 rearrangement and BRAF V600E mutations and one of them died 15 days after taking dabrafenib, while the other one died 11 days after taking dabrafenib and trametinib (35, 36). The third patient who developed BRAF V600E secondary to ROS1 rearrangement loss on crizotinib received a partial response of more than 6 months with dabrafenib and trametinib (37).

To date, two-drug therapy is only approved in NSCLC with BRAF V600E for FDA indication and recommended by the NCCN guideline. However, some studies showed that the treatment mode and clinical characteristics of BRAF V600E were similar with other subtypes, such as BRAF V600K (58). In light of guidelines for BRAF V600 mutated melanoma, dual-targeted therapy is also recommended. Therefore, this review recommends that dual-targeted therapy (dabrafenib and trametinib) could be initiated in BRAF V600 mutated patients, as a congener disease as well. Now that we know the treatment for BRAF V600, and then we talk about how to treat BRAF non-V600?

### Targeting BRAF Non-V600

The most patients of BRAF non-V600 mutation has less aggressive phenotype and significantly superior survival compared to those with BRAF V600 mutation, suggesting the potential need of different therapeutic strategies (59). A retrospective multicenter cohort study concluded that patients with BRAF non-V600E mutations located outside of the activation segment of the BRAF kinase domain were resistant to BRAF therapy (60). Another trial recommended chemotherapy as the dominant strategy for non-V600 mutation patients in the first-line treatment (61).

Recent experiences *in vitro* and *in vivo* show that class IIa BRAF mutant cells were sensitive to single-agent BRAF inhibitors, whereas class IIb BRAF mutant cells were not (62). Moreover, dual MAPK pathway inhibition (dMAPKi) effectively impaired the growth of subsets of non-V600 (62). *In vitro*, other trials have also demonstrated that BRAF non-V600 (L597, K601E) had significant response to MEK inhibitors (63).

Instead, research about class III mutant that have impaired kinase activity or are kinase-dead and linked with high RAS levels suggest Class III BRAF mutants may be treated with MEK inhibitors which co-existing with mutations in *RAS* and NF1 in melanomas, but in epithelial tumors, the great majority of class III mutations are not associated with RAS/NF1 alterations and may be treated with receptor tyrosine kinase (RTK) inhibitors that block the RAS pathway (20). Another study came to similar conclusions (64). A case report have also shown that dMPAKi is also benefit for patients harboring a dual G469A and W604C BRAF mutations and the response is more than 15 months (65). However, other studies found vemurafenib is not effective in NSCLC patients with BRAF non-V600 mutation (44, 66).

There is no evidence for patients resisted to EGFR-TKI yet, which could result in BRAF non-V600 mutations. A basic research demonstrated that, in osimertinib resistant PC9 cells transfected with BRAF G469A mutant plasmid, the combination of osimertinib, selumetinib (MEK 1/2 inhibitor) and trametinib (MEK 1/2 inhibitor) or dabrafenib reversed osimertinib resistance (67). Except for the two classical mutations of BRAF V600 and non-V600, there are also co-mutations of BRAF, and we will continue to discuss the treatment of BRAF co-mutations.

### Treatment Recommendations for BRAF Co-Mutations

There is no high-level clinical trial for primary BRAF co-mutations to date. Since BRAF co-mutations were a clinical problem, we provide some recommendations for reference. Based on the studies on EGFR/ALK co-mutations, the phosphorylation level of the mutant genes would be a rational treatment option, and the abundance of gene mutations was also a positive biomarker for clinical decision (68). Besides, the treatment of primary BRAF co-mutation can refer to the treatment of secondary BRAF activation with EGFR/ALK/ROS1 mutation and adopt double or triple targeted therapy (53, 69). Considering the cost effectiveness and adverse events, immunotherapy, specifically immune checkpoint inhibitors (ICIs) would be a choice for BRAF co-mutations, which is introduced in detail as follows. We have solutions for all three of BRAF mutation patterns. And in recent years, the rise of immunotherapy has also brought new solutions to BRAF mutations.

### Immunotherapy

Studies have reported BRAF mutant NSCLC patients have high expression of programmed cell death ligand 1 (PD-L1), which means that patients with BRAF mutation have great potential for ICIs (4). A retrospective cohort study conducted in 31 NSCLC patients with BRAF mutations showed that there was no statistically significant difference in OS among BRAF classic mutant patients who received first-line chemotherapy or immunotherapy (70). In a multi-institution retrospective chart review of 39 patients with BRAF mutated NSCLC, 22 of whom received ICIs, the ORR for V600E and non-V600E were 25% and 33%, respectively (*P* =1.0); PFS was similar in patients received ICIs treatment; median OS was equal for patients who received or did not receive ICIs (71). Another study collecting 4178 patients and 4462 samples from a cBioPortal database showed that BRAF wild-type mutants had a longer OS than BRAF mutants. Unlike previous study, this study showed that non-V600E had a longer OS than V600E under ICIs treatment (72). A BRAF G469A mutant NSCLC case obtained a deep and durable response after ICIs treatment, which suggested that BRAF non-V600 mutation may benefit more from immunotherapy than EGFR/ALK-driven mutation in NSCLC (73). However, in a retrospective, multicenter and real world analysis, 44 of 107 patients with BRAF mutations (V600:26, non-V600:18) received ICIs, with the response rates of 26% in BRAF V600 cohort and 35% in the non-V600 cohort. Besides, BRAF V600 cohort have longer PFS and OS than non-V600 cohort (74).

The above studies demonstrate the survival in various types of BRAF mutations treated with ICIs immunotherapy or targeted therapy are different. However, there is a lack of high-level evidence to prove which is better. Further prospective clinical trials are necessary to prove which is the optimal first line strategy. We have solutions for all three of BRAF mutation patterns. In recent years, the rise of immunotherapy has also brought new solutions to BRAF mutations.

### Strategies for Resistance to BRAF Inhibitors

BRAF mutant tumors might initially respond to treatment with BRAF inhibitors, but eventually developed drug resistance. For acquired resistance to BRAF inhibitors caused by BRAF fusion, clinical trials have demonstrated the efficacy of pan-RAF inhibitors in patients with BRAF fusion (75). Evidence suggests that BRAF proteins undergo homodimerization and heterodimerization, therefore BRAF rearrangement is very insensitive to BRAF inhibitors. And RAF inhibitors could bind and inhibit all RAF isomers, so they are effective for BRAF fusion (76, 77). Evidence is also provided that a combination of MEK inhibitors and EGFR inhibitors is effective in patients with BRAF fusion (25, 77). As for acquired drug resistance, a series of post-resistance measures were reported. Intermittent dosing would be a choice. A melanoma case with vemurafenib showed the accelerated growth of RAS-mutant leukemia, and intermittent dosing of vemurafenib relieved the disease and reduced the disease burden (78). Subsequent studies showed that intermittent dosing of BRAF inhibitors and RAF inhibitors may delay the progression of resistant tumors and make it sensitive to inhibitors again (79, 80). An international team of 180 scientists proposed the concept of a low toxicity “broad-spectrum” treatment based on the sequencing of the cancer’s genome, which targeted multiple key tumorigenesis pathways and mechanisms to prevent cancer growth (81). What’s more, for resistant mutations at different gene target, different drug combinations could be adopt. For example, FDA has approved the combination of BRAF inhibitors (Vemurafenib and Dabrafenib) and MEK inhibitor trametinib for the treatment of BRAF inhibition resistance. And clinical studies of PI3K/AKT inhibitor plus MAPK inhibitor, everolimus (RAD001) plus bevacizumab, everolimus (RAD001) plus temozolomide (TMZ), and targeted therapy plus immunotherapy have also been conducted, but there is a lack of more data to support these therapies, so further exploration is needed (82). The strategies for resistance to BRAF inhibitors were listed in **Table 1**.

**Table 1:** Strategies for resistance to BRAF inhibitors.

Situation	Strategies	Ref.
BRAF fusion	pan-RAF inhibitors	(75)
	BRAF inhibitors and RAF inhibitors	(76, 77)
	combination of MEK inhibitors and EGFR inhibitors	(25, 77)
Acquired drug resistance to vemurafenib	intermittent dosing	(78–80)
Changes in multiple key tumorigenesis pathways and mechanisms	low toxicity “broad-spectrum” treatment	(81)
Resistant mutations at different gene target	different drug combinations	(82)

## The Evolution of Braf Activation

### The Pathways of BRAF Evolution

**Figure 2:**
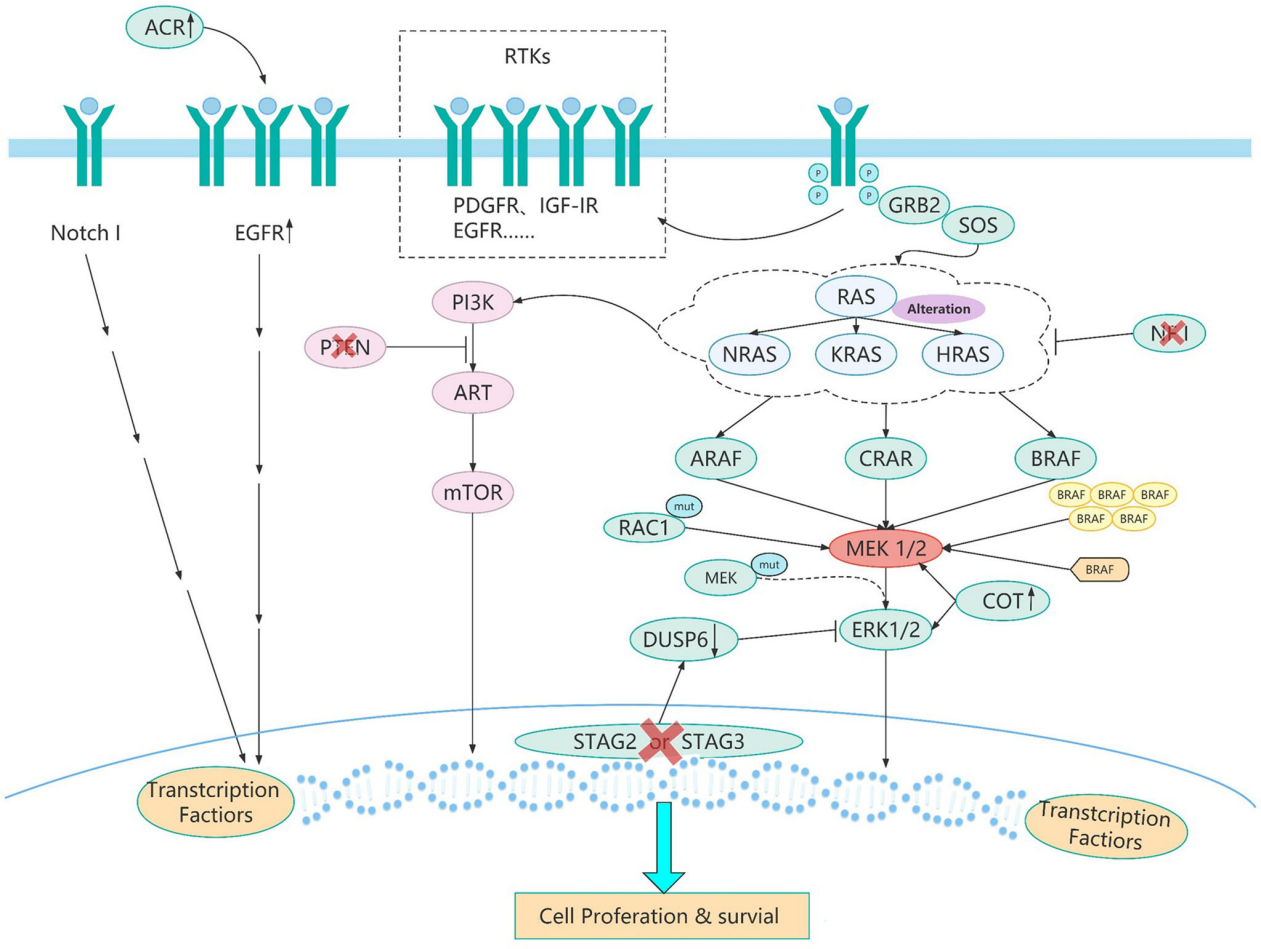
The resistance pathways of BRAF inhibitor. BRAF mutated tumor cells evolve different drug resistance pathways to maintain cell growth after chronic inhibition by BRAF inhibitors. These evolutionary mechanisms (**Table 2**) include BRAF splice variants, BRAF copy number amplification,CRAF overexpression, MEK1 mutations, and other mechanisms. Different pathways of BRAF evolution can tell us how to overcome the problem of resistance to BRAF inhibitors and how to develop more rational protocols to address the resistance problem.

As reported, the evolution of BRAF comes from the changes of various genes mainly in the following three ways after targeted therapy (**Figure 2**). First, the changes of BRAF itself and BRAF downstream molecules which lead to resistance to BRAF targeted inhibitors mainly come from the following aspects. BRAF splice variants are the most common situation. Studies has demonstrated that AGK-BRAF fusion leads to loss of the CR1 region of BRAF, thereby eliminating the inhibitory RAS-binding domain, and results in RAS-independent constitutive activation of the kinase (83). A research has found that the loss of the inhibitory RAS binding domain resulting from the loss of the internal BRAF leads to the reactivation of RAS-RAF-MEK-ERK signaling and mediates resistance to BRAF inhibitors (23). Besides, BRAF copy number amplification also can lead to resistance to BRAF targeted inhibitors. Hubing Shi et al. (84) proved that BRAF V600E amplification was the mechanism of acquired resistance of BRAF inhibitors, providing evidence for drug target changes leading to clinical relapse. Moreover, Montagut et al. (85) found that CRAF overexpression to increased ERK1/2 level indicating some BRAF mutant tumor cells were primary insensitive to RAF inhibition in the experiment, which was related to a switch from BRAF to CRAF dependency in tumor cells. And Lu et al. (86) found p21-activated kinases phosphorylate CRAF and MEK to reactivate ERK, which drive acquired drug resistance to MAPK inhibitors in BRAF mutants. Furthermore, MEK1 mutation can also lead to reactivation of the MAPK pathway. MEK is downstream of RAS signaling MEK reactivation caused by MEK mutation does not require stimulation of BRAF signaling, so BRAF inhibitors are ineffective against MEK1/2 mutation. Therefore, MEK1 mutation can promotes ERK phosphorylation, and MEK2 can also heterodimerize with MEK1, ultimately leading to the reactivation of EKR (87).

Second, changes in upstream molecules of BRAF lead to the evolution of BRAF mainly from the following aspects. First of all, studies have shown that NRAS upregulation is another resistance mechanism of BRAF inhibitors and NRAS upregulation may promote the dimerization of RAF, which will cause insensitivity of ERK signaling to drugs, leading to tumor drug resistance (88–90). And the mutation of RAS gene may lead to the reactivation of MAPK pathway. On the one hand, the mutant RAS protein will not dissociate after binding to GTP but become permanently activated. On the other hand, overactivated RAS may lead to overactivation of ARAF and CRAF, and thus cell proliferation. These two aspects jointly promote signal transduction of MAPK pathway (89, 91, 92). And ERK protein is a negative regulator of RAS protein, BRAF inhibitors can inhibit ERK pathway, thereby inducing part of RAS activity and leading to the activation of MAPK pathway (93, 94). And as well as RTKs alteration, overexpression of platelet derived growth factor receptor (PDGFR)-β or siRNA knockdown of PDGFRβ demonstrates the potential role of PDGFRβ signaling in drug resistance, and the introduction of PDGFRβ into untreated cells reduces sensitivity to vemurafenib (89). In addition, up-regulation of EGFR expression was found in BRAF inhibitor resistant cell lines and resistant tumor biopsies (95). EGFR activation binds to specific tyrosine residues on the receptor and results in a conformation change of Sos protein, thereby recruiting and activating RAS-GDP, and finally ERK activation induces cell proliferation (96). Besides, the upregulation of IGF1R/IR in BRAF and MEK inhibitor resistant cells and the maintenance of P-ERK and P-Akt suggest that IGF1R/IR may mediate resistance to inhibitors through the reactivation of MAPK (97).

Third, activation of bypass pathways leads to overactivation of the BRAF signaling pathway mainly come from the following aspects. At first, elevate expression of COT, like CRAF, activates ERK through MEK-dependent mechanisms that do not require RAF signals, thus driving resistance to RAF inhibition (98). Besides, studies have shown that loss of STAG2 or STAG3 inhibits CCCTC-binding factor (CTCF) mediated dual specificity phosphatase 6 (DUSP6) expression, leading to a significant decrease in DUSP6 protein levels and ultimately reactivation of MEK-ERK signaling in BRAF-inhibitor treated melanoma cells (99). What’s more, RAC1 is a GTP-binding protein that modulates cytoskeletal rearrangement by signaling g-protein-coupled receptors and other molecules and RAC1 P29S mutations may mediate resistance to vemurafenib and dabrafenib by maintaining MAPK signaling (100). Additionally, NF1 is a tumor suppressor that inhibits RAS activity. Experiments have proved that loss of NF1 can re-drive MAPK pathway by activating RAS activity and increasing CRAF, thus mediating resistance to RAF inhibitors (101). Moreover, studies have found that ACK1 can inhibit the expression of EGFR, so the loss of ACK1 induces the increase of EGFR protein, thus increasing cell signal transduction to mediate the generation of drug resistance (102). Furthermore, it has been confirmed that increased Notch signaling results in increased expression of markers associated with cell dedifferentiation and increased cell migration and does not reactivate ERK in the presence of drug therapy to mediate acquired resistance to MAPK inhibitors (103). Finally, the study of Hubing Shi et al. (56) named the PI3K-PTEN-AKT pathway as the second core resistance pathway, and it has been reported that upregulation of the PI3K pathway accounts for approximately 22% of BRAF inhibitor acquired resistance melanoma. PTEN is an important tumor suppressor, which acts to counteract the effect of PI3K and when PTEN is lost, mutant or methylated, the activity of PI3K pathway will increase, and cells can finally survive by adopting PI3K signal (104). The evolution and pathways of BRAF activation were listed in **Table 2** and **Figure 2**. We have collected several paths of BRAF evolution. In addition, we hope to find certain rules from the evolutionary pathway, so we need to study the pathway of BRAF evolution through some methodologies.

**Table 2:** The evolution and pathways of BRAF activation.

Cancer types	Evolutionary types	Evolutionary pathways	Ref.
Melanoma	changes in BRAF itself	BRAF splice variants	(23, 83)
Melanoma	**-**	BRAF copy number amplification	(84)
Melanoma	downstream of the BRAF	CRAF overexpression	(85, 86)
Melanoma	**-**	MEK1 mutations	(87)
Melanoma	upstream of the BRAF	RAS alteration	(88, 94)
Melanoma	–	RTKs alteration	(89, 95, 97)
Melanoma	activation of bypass pathways	Elevated expression levels of COT	(98)
Melanoma	–	Loss of stromal antigen 2 (STAG2) or STAG3	(99)
Melanoma	–	RAC1 mutation	(100)
Melanoma	–	Loss of NF1	(101)
Melanoma	–	Loss of ACK1	(102)
Breast cancer and melanoma	–	Activation of the Notch1 pathway	(103)
Melanoma	–	Phosphoinositide 3-kinase (PI3K)/AKT pathway dysregulation	(56, 104)

### Methodology to Track the BRAF Evolution

Due to the diversity and randomness of gene evolution, we need to use various emerging technologies and methods to find certain rules from dynamic evolution, so as to obtain certain therapeutic effects, and also to find effective therapeutic strategies. With the advent of cancer genomics and the development of multi-region sequencing, single-cell correlation sequencing and cloning techniques, it has become possible to describe gene phylogeny and evolution (105–107). In recent years, studies have been carried out on clonal phylogeny using single time point snapshot, multi-region sampling and spatio-temporal modeling to analyze diseases. In addition, mathematical models and other methods can be used to explore new evolutionary methods. At the same time, it also puts forward the direction and challenge to bioinformatics and computer science (108). In addition, it has been proposed that the development of single-cell multi-omics technology is crucial for a comprehensive understanding of the evolutionary mechanism. For example, multiple sampling methods can be used to analyze the evolutionary mechanism of tumors by different sampling methods (such as multiple regions or multiple times). And examples include *in vivo* and *in vitro* modeling of tumor evolution through optical or sequencing barcodes (109). Furthermore, deep sequencing of multiple regions of a tumor directly to detect evolutionary mutations is another way (110).

## Undergoing Studies for Braf Activation

The efficacy and safety of BRAF inhibitors are being explored in several clinical studies (e.g., NCT03915951, NCT04543188 etc.). In addition, more treatment options for patients with BRAF mutations can be explored, for example, BRAF inhibitors as adjuvant/neoadjuvant therapy for patients with NSCLC; BRAF inhibitor combined with MEK inhibitor and EGFR-TKI as three-target combination therapy; BRAF inhibitors combined with immunotherapy, anti-angiogenic drugs and other drug combinations. With the success of the ADAURA study, a new direction of targeted therapy in the adjuvant treatment for NSCLC patients has been opened. Therefore, we believe that the use of dabrafenib in combination with trametinib in neoadjuvant/adjuvant therapy for early-stage NSCLC patients is worthy of further exploration (111). Besides, for patients with acquired resistance to BRAF inhibitors, re-biopsy and NGS test to find new targeted drugs or new combination therapy are necessary. Finally, we still want to know if the strategy of dual-targeted or triple-targeted therapy could be re-challenged. Small-sample case reports suggest that sequential therapy with targeted therapy and immunotherapy, combined with the “rechallenge” of dabrafenib and trametinib, may benefit patients with V600E mutation and positive PD-L1 (112). Among the 2 patients in the prospective match-R study, 1 patient was switched to chemotherapy and then dual-target therapy after double-target drug resistance. Another patient, after double-target drug resistance, was first switched to immunotherapy, followed by chemotherapy, and then sequential double-target therapy, all of which achieved disease stability in the “re-challenge” of double-target therapy (113). These explorations are expected to become hot research directions in the future, and we eagerly look forward to more effective drugs or treatments. The undergoing studies for BRAF activation were listed in **Table 3**.

**Table 3:** Selected ongoing trials with BRAF Inhibitors for NSCLC.

Clinical Trial Identifier	Study Design	Intervention/s	Setting	Primary Endpoint	Phase	Status
NCT03915951	90 participants Open-label, Multicenter, Non-randomized, Phase 2 study	Encorafenib plus Binimetinib	First line	ORR	Phase 2	Recruiting
NCT04543188	225 participants two-part, phase 1A/B, open-label, multicenter trial evaluating pharmacokinetics	PF-07284890 plus Binimetinib plus Midazolam	First line	DLTs, AEs, Overall response	Phase 1	Recruiting
NCT04526782	119 participants Open-label, Multicenter, multi-cohort Phase 2 study	encorafenib plus binimetinib	First line	ORR	Phase 2	Recruiting
NCT05003622	6 participants Multicenter, Open-label, Phase 1 Study	Encorafenib	First line	DLTs	Phase 1	Active, not recruiting
NCT05065398	20 participants Open Label, Multicenter Phase II Clinical Trial	HLX208	First line	ORR	Phase 2	Recruiting
NCT05275374	221 participants Dose-escalation and Expansion Phase I/IIa Study	XP-102 or XP-102 plus Trametinib or	First line	Characterize the safety of XP-102, Evaluate the pharmacokinetics of XP-102, Establish maximum tolerated dose of XP-102	Phase 1 Phase 2	Not yet recruiting
NCT05195632	55 participants Multicenter, Open-label, phase 2 study	Encorafenib plus Binimetinib	First line	DLTs, ORR	Phase 2	Not yet recruiting
NCT02974725	331 participants Phase Ib, Open-label, Multicenter Study	LXH254 plus LTT462 or LXH254 plus Trametinib or LXH254 plus Ribociclib	First line	DLTs, AEs, Tolerability	Phase 1	Recruiting
NCT04620330	100 participants Multicenter, Non-randomized, Open-label Phase 1b/2 study	VS-6766 or VS-6766 plus Defactinib	First line	the optimal regimen, the efficacy of the optimal regimen	Phase 2	Recruiting

## Conclusions

There are many forms of activation of BRAF in primary and secondary activation, including classical mutations (BRAF V600 and non-V600), other mutations, BRAF fusion, rearrangement, in-frame deletions, insertions, co-mutations, etc. with different biological phenotypes, medical senses and different subsequent treatments. Currently, the FDA recommends dual targeted drug combination for BRAF V600, while there is no unified treatment regimen for other types of BRAF mutations. As for the treatment of primary BRAF co-mutations, it could base on a comprehensive consideration of the phosphorylation level and abundance of the mutant genes, cost effectiveness and adverse events of combined targeted therapy. Immunotherapy can also benefit for patients with BRAF mutations with high PD-L1 expression in small sample size studies. After resistance of BRAF inhibitors, the evolution of BRAF mainly evolves through activation of upstream, downstream and bypass pathways of BRAF. The evolutionary pathway can be tracked by various emerging technologies including genomics, next-generation sequencing, single-cell sequencing and cloning techniques, which may find a solution for the resistance of BRAF inhibitors.

In the future, it’s necessary to explore head to head clinical trials to compare targeted therapy with immunotherapy, to develop drugs for other BRAF mutations except V600, to find new strategies for the resistance of BRAF inhibitors. Furthermore, whether BRAF inhibitors can be used as adjuvant/neoadjuvant therapy or re-challenged treatment are likely to be hot topics.

## Author Contributions

LYZ and JS researched the data, wrote the review, and designed the figure. JGS, LPZ, and QY reviewed and revised the manuscript. All authors contributed to the article and approved the submitted version.

## Funding

This study was supported by the National Natural Science Foundation of China (81773245, 81972858, 82172670), the Chongqing Innovation Leading Talents Program (cstccxljrc201910) and the Cultivation Program for Clinical Research Talents of Army Medical University in 2018 (2018XLC1010).

## Conflict of Interest

The authors declare that the research was conducted in the absence of any commercial or financial relationships that could be construed as a potential conflict of interest.

## Publisher’s Note

All claims expressed in this article are solely those of the authors and do not necessarily represent those of their affiliated organizations, or those of the publisher, the editors and the reviewers. Any product that may be evaluated in this article, or claim that may be made by its manufacturer, is not guaranteed or endorsed by the publisher.
